# Nano Metal-Containing Photocatalysts for the Removal of Volatile Organic Compounds: Doping, Performance, and Mechanisms

**DOI:** 10.3390/nano12081335

**Published:** 2022-04-13

**Authors:** Rong Cheng, Jincheng Xia, Junying Wen, Pingping Xu, Xiang Zheng

**Affiliations:** 1 School of Environment and Natural Resources, Renmin University of China, Beijing 100872, China; chengrong@ruc.edu.cn (R.C.); 18813071017@163.com (J.X.); 2017101003@ruc.edu.cn (J.W.); 2 School of Architectural Equipment Engineering, Zhejiang College of Construction, Hangzhou 311231, China

**Keywords:** photocatalysts, VOCs, doping, photocatalytic oxidation, mechanisms

## Abstract

Volatile organic compounds (VOCs) in indoor air are considered a major threat to human health and environmental safety. The development of applicable technologies for the removal of VOCs is urgently needed. Nowadays, photocatalytic oxidation (PCO) based on metal-containing photocatalysts has been regarded as a promising method. However, unmodified photocatalysts are generally limited in applications because of the narrow light response range and high recombination rate of photo-generated carriers. As a result, nano metal-containing photocatalysts doped with elements or other materials have attracted much attention from researchers and has developed over the past few decades. In addition, different doping types cause different levels of catalyst performance, and the mechanism for performance improving is also different. However, there are few reviews focusing on this aspect, which is really important for catalyst design and application. This work aims to give a comprehensive overview of nano metal-containing photocatalysts with different doping types for the removal of VOCs in an indoor environment. First, the undoped photocatalysts and the basic mechanism of PCO is introduced. Then, the application of metal doping, non-metal doping, co-doping, and other material doping in synthetic metal-containing photocatalysts are discussed and compared, respectively, and the synthesis methods, removal efficiency, and mechanisms are further investigated. Finally, a development trend for using nano metal-containing photocatalysts for the removal of VOCs in the future is proposed. This work provides a meaningful reference for selecting effective strategies to develop novel photocatalysts for the removal of VOCs in the future.

## 1. Introduction

Volatile organic compounds (VOCs) are defined as organic compounds with a low initial boiling point (≤260 °C) at standard atmospheric pressure and can exist in the environment as gases [1]. It is proven that VCOs are widely sourced and generally exist in the environment. VOCs in the environment, including alkanes, alkenes, alkynes, aromatic hydrocarbons, as well as organic compounds containing nitrogen, oxygen, sulfur, and halogens, threaten the environment and human health [2]. VOCs may damage the respiratory system, lung function, nervous system, endocrine system, increase the risk of genetic mutations, resulting in allergies, chronic respiratory diseases, cancer, and other diseases [3]. Specifically, benzene and toluene, the main components of VOCs in indoor air, can directly enter the human body through the respiratory tract and skin, causing diseases of the human respiratory, blood, liver, and other systems [4]. Besides, VOCs could also react with the ozone, resulting in multiple hazardous by-products [5], and participate in the reactions with NO_x_ (NO, NO_2_), leading to the formation of O_3_, peroxyacetyl nitrate (PAN), and other oxidization species [6]. Therefore, it is of great significance to develop effective VOC removal technology to control air pollution and protect human health.

Over the past few decades, several cleaning techniques including ventilation [7], adsorption [8], biological treatment [9], gas separation [10], and chemical oxidation [1] have been proposed for the removal of VOCs, but most of these techniques are limited by their insurmountable shortcomings. For example, ventilation is usually limited by efficiency, time, and outdoor air quality [7]; absorption requires adsorbents with high adsorption capacity and high regeneration ability [11]; biological treatment has high requirements for strain screening and culture; and gas separation and chemical oxidation are difficult to apply in indoor environments due to their high cost and operating conditions [12]. Nevertheless, photocatalytic oxidation (PCO) is considered as one of the most promising technologies for the removal of VOCs, owing to its low cost, simple operation, less reaction restriction, and complete degradation [4,13,14,15]. The PCO process can degrade VOCs directly into H_2_O and CO_2_, instead of mere adsorption, separation, and disposal [16].

The PCO process for the removal of VOCs is based on the principle that radiation of light can be absorbed by semiconductor photocatalysts, which drives the formation of radicals and reactive oxygen species (ROS) to decompose VOC molecules [13]. Several mechanisms for the decomposition of air contaminants including VOCs have been proposed. The dominant view is that reactive oxygen species (ROS)/hydroxyl radicals (or some other equivalent oxidant) are photo-generated on the surface of photocatalysts. The oxidation capacity of such oxidants is the reason for degradation of various pollutants in the environment, including toluene [17] and formaldehyde [18] in the air.

In PCO, the removal of VOCs is conducted by photocatalysts under UV/Vis light [17]. Photocatalysts play an important role in the PCO process, which largely determines the removal effect of VOCs. Since the pioneering use of TiO_2_ for degradation of pollutants in the environment, a large number of PCO photocatalysts have been developed and proven to be effective [19]. The common photocatalysts in PCO are a class of metal-containing semiconductors such as TiO_2_, WO_3_, ZnO, ZnS, Fe_2_O_3_, CdS, and SrTiO_3_ [20]. Nowadays, some metal-free and organic photocatalysts have been developed for PCO processes, but they generally have lower photocatalytic activity compared with metal-containing photocatalysts [21]. In the past few decades, a great deal of research has been devoted to developing efficient and stable metal-containing photocatalysts for the removal of VOCs in indoor air and numerous modification strategies have been proposed, such as doping, nanostructure, crystal regulation, and heterojunction [21,22,23]. Doping is a relatively simple and effective strategy for the synthesis of efficient photocatalysts. Doping other materials or elements can regulate the band structure of photocatalysts and improve the activity of the photocatalysts [8,22]. Nanotechnology has been widely applied in the synthesis of photocatalysts with high catalytic activity and unique nanostructures, such as nanoparticles [24], nanotubes [25,26,27], nanofibrous mats [28], nanobelts [29], and nanofibers [30]. These stable nano photocatalysts have strong photocatalytic activity under UV/Vis light. In addition, common nano photocatalysts are generally recognized as safe materials, mild oxidants, and the required ambient temperature is not rigorous [31].

With great attention attached to air pollution around the world, the application of nano metal-containing photocatalysts in the removal of VOCs has aroused great interest and made considerable developments in recent years [2]. Existing work has mainly focused on the mechanisms of photocatalysts, the PCO process, and key influencing factors; however, there are few reviews on photocatalyst modification and the associated performance and mechanisms. A review of the modification strategies and related performance of photocatalysts is necessary because it is important to apply appropriate strategies for the synthesis of photocatalysts for the removal of VOCs in indoor air. Here, we give a comprehensive review on the nano metal-containing photocatalysts: doping, performance, and mechanisms. First, the common unmodified metal-containing photocatalysts applied in removal of VOCs and basic mechanisms are introduced. Then, the synthesis methods, performance, and mechanisms of nano metal-containing catalysts doped with metal and/or non-metal or other materials are reviewed based on the latest progress. At last, the limitations of the existing research are proposed and prospects for future development are put forward by the authors of this article. This work can provide useful guidance for the synthesis and modification of photocatalysts for the removal of VOCs in indoor air.

## 2. Undoped Metal-Containing Photocatalysts for the Removal of VOCs

In PCO, the removal of VOCs is conducted by photocatalysts under UV/Vis/IR light at room temperature [17,32]. The major types of semiconductors used for the removal of VOCs include oxides, binary chalcogenide metal semiconductors, multivariate metal semiconductor catalysts, metal-free materials [33], and others [11,34]. Oxides and binary chalcogenide metal semiconductors are the most widely studied photocatalysts with high removal efficiency and have been applied in practice in the past few years [21]. These photocatalysts are generally oxides or sulfides of transition metals, including TiO_2_, ZnO, CeO_2_, ZrO_2_, WO_3_, α-Fe_2_O_3_, CdS, and ZnS [35].

Band gap is an important parameter of photocatalysts, which determines the light response range and catalytic activity of the photocatalyst. The dominant view of the mechanism is that the electronic band structure of photocatalysts comprises a valence band (VB) occupied by electrons and a conduction band (CB) without electrons. The two bands are separated by a band gap, forbidding the electrons to escape from the VB [13]. Equations (1)–(8) describe the mechanism of the PCO process for VOCs degradation using photocatalysts. The conductive process will be stimulated when the light provides enough energy for the electron (e^−^) in VB and the electron elevates to the vacant CB by breaking through the band gap, which also generates an equivalent hole in VB (h^+^), as shown in Equation (1). The energy of light generally requires a range of 3.0–5.0 eV, which is for the electrons across the energy barrier between the two bands. Traditional photocatalysts are normally activated by UV light, while recent reports have developed novel photocatalysts utilizing Vis/IR light as excitation energy [17,32].

Catalyst + *hv* → h^+^ + e^−^ (1)

O_2_ + e^−^ →·O_2_^−^ (2)

H_2_O + h^+^ →·OH + H^+^ (3)

·O_2_^−^ + H^+^ → HO_2_ (4)

HO_2_·→ O_2_ + H_2_O_2_ (5)

HO_2_· + H_2_O +e^−^ → H_2_O_2_ + OH^−^ (6)

·OH + ·OH → H_2_O_2_ (7)

H_2_O_2_ + e^−^ → ·OH + OH^−^ (8)

The generally accepted model of PCO [36] indicates that the electrons and holes diffuse to the surface of the photocatalysts and react with the VOCs. However, the alternate path of electrons and holes is to recombine before decomposing the VOCs, which clearly undermines the efficiency of VOC decomposition. When the photo-generated electrons and holes diffuse to the surface and do not recombine, these electrons and activated holes (h^+^) are responsible for driving redox reactions of VOCs adsorbed on the surface of photocatalysts [34].

The electrons generated from photocatalysts could react with electron acceptors, such as O_2_ (Equation (2)), and the holes could oxidize the electron donors, such as H_2_O, driving the formation of radicals (Equation (3)). When radicals (·OH) escape from the surface of photocatalysts, they can lead to the formation of reactive oxygen species (ROS) (Equations (2)–(8)), which can also react with the VOCs because of the unsaturated bonds. The radicals and ROS generally include ·OH, ·O_2_^−^, HO_2_, and H_2_O_2_. They eventually oxidize VOCs into CO_2_, H_2_O, and other products.

Table 1 lists the band gap of common metal-containing photocatalysts. ZrO_2_ has the high band gap (5.00 eV), which means that photoexcitation needs to absorb higher energy photons. TiO_2_ and ZnO with lower band gap (3.02~3.37 eV) can use light in a larger wavelength range. Although WO_3_, CdS, and α-Fe_2_O_3,_ have a relatively lower energy band gap (2.09~2.75 eV) compared with other catalysts (>3.0 eV), which means that they could utilize a larger range of wavelength of solar spectrum, this type of photocatalyst usually has a high carrier recombination rate, so it cannot be directly used for the degradation of VOCs.

The difference of the decomposition performance of diverse photocatalysts’ VOCs partly depends on the momenta types of charge carriers. When the momenta of electrons and holes are equal, the energy of the inlet light is equal to the energy band gap. This type is considered as the direct type, such as TiO_2_ [37], ZnO [38], SnO_2_ [41], and WO_3_ [43]. Alternately, the other type is considered as the indirect type, in which the two charge carriers have different momenta, leading to the fact that the conservation of momenta is larger than the band gap, which indicates the inlet light energy is surplus and requires crystal lattice to transfer. The indirect cases are TiO_2_ [37], CeO_2_ [42], ZrO_2_ [39], and α-Fe_2_O_3_ [45]. Experimental and practical cases both show that the indirect type of catalyst requires lower inlet light energy and lower rates of generation of recombination of electrons and holes.

The photocatalytic decomposition mechanism of VOCs has been extensively studied. Common VOCs (formaldehyde, benzene, toluene, etc.) react in different steps with radicals and ROS, and they eventually degrade to inorganic matter in air. Formaldehyde (HCHO) is the most widely researched one because of its widespread presence in the air. Additionally, HCHO is carcinogenic, toxic, and hard to decompose in the environment. TiO_2_ has high photocatalytic efficiency under UV in terms of the destruction of HCHO [46]. Equations (9)–(16) show how HCHO reacts with the radicals and ROS in the presence of a TiO_2_-based photocatalyst. The active hydroxyl radical (·OH) and superoxide anion radical (O_2_^−^) act as the oxidation together, first oxidizing formaldehyde to hydroxy acid, and finally decomposing it into CO_2_ and H_2_O. The degradation mechanism is presumed as follows, the ·OH extracts hydrogen from formaldehyde to form the ·CHO; and the ·CHO can be further oxidized to a carboxylic acid in two ways; then, the carboxylic acid is further oxidized and decomposed into CO_2_ and H_2_O. In fact, for some difficult-to-degrade VOCs such as benzene and toluene, the PCO process often cannot directly mineralize them into CO_2_ and H_2_O, but ends in corresponding acids, aldehydes, and ketones [47].

HCHO + ·OH ⟶·CHO + H_2_O (9)

·CHO + ·OH ⟶ HCOOH(10)

·CHO + ·O_2_^−^ ⟶ HCO_3_ + H^+^ ⟶ HCOOOH (11)

HCOOOH + HCOH ⟶ HCOOH(12)

HCOOH ⟶ HCOO^−^ + H^+^ (13)

HCOO^−^ + ·OH ⟶ H_2_O + CO_2_ (14)

HCOO^−^ + h^+^ ⟶ H^+^ + ·CO_2_^−^ (15)

CO_2_^−^ + h^+^ ⟶ CO_2_ (16)

Due to the nanoscale effect, nano photocatalysts facilitate migration of internal photo-generated e^-^ and h^+^ to the surface of the catalysts in the reaction. Nano-sized catalysts usually have a higher specific surface area that helps to absorb more VOC molecules and provide more reaction sites, so the reaction activity is improved [27,48]. Therefore, nanotechnologies are usually used to synthesize photocatalysts with specific nanostructures, which have higher catalytic activity than the bulk structure [49]. Nanoparticles are the most important form of photocatalysts, such as TiO_2_ nanoparticles, TiO_2_/SiO_2_ nanoparticles, SiTiO_3_ nanoparticles, CeO_2_ nanoparticles, etc. These nanoparticles have a higher specific surface area, more reaction sites, can absorb more VOCs molecules in the reaction, and generate more radicals and ROS to participate in the reaction. Besides, nano photocatalysts in the form of nanotubes, nano-films, nano-fibers, and nano-belts photocatalysts are also applied in the removal of VOCs. These unique nanostructures can optimize the energy band structure, specific surface area, pore size, and other properties of the photocatalyst to improve the activity, and have good degradation effects on alkanes, formaldehyde, acetaldehyde, benzene, toluene, and some chlorinated volatile organic compounds [26].

Some nano metal-containing photocatalysts used for the removal of VOCs, including binary chalcogen compound photocatalysts and their composites combined with adsorbent materials are shown in Table 2. The most popular one is TiO_2_ anatase, which has been commercialized. TiO_2_ is relatively inexpensive and chemically stable. It also has highly redox reaction abilities and requires no chemical additives [18]. There are three polymorphs of TiO_2_, that is, anatase, rutile, and brookite. In fact, different polymorphs of TiO_2_ perform differently in decomposing VOCs. It is commonly accepted that anatase is the most effective form, due to its longer recombination time of two charges carriers and higher density of surface hydroxyl radicals [50].

TiO_2_ nanoparticles are usually synthesized by flame spray pyrolysis and the sol-gel method, and have a good removal effect on a variety of gaseous pollutants. Maira, A.J et al. [48] synthesized a series size of TiO_2_ nanoparticles by the sol-gel method. They pointed out that the particle size of TiO_2_ affected its specific surface area rather than its aggregation morphology. The smaller nanoparticles offer higher specific surface area. TiO_2_ nanoparticles with a diameter of 7 nm have the highest degradation efficiency of trichloroacetic acid. Larger or smaller sizes of nanoparticles will reduce the degradation efficiency. The decrease in catalytic efficiency of crystal sizes smaller than 7 nm may be due to changes in the nanocrystalline structure and elevator characteristics. In addition, other gases in the environment will also affect the removal of VOCs. Ao, C.H. et al. [18] explored the influence of NO, SO_2_, benzene, toluene, ethylbenzene, and xylene (BTEX) on the removal efficiency of formaldehyde catalyzed by p25 under typical indoor formaldehyde concentrations (PPb). The research showed gaseous water and NO could promote the oxidation of formaldehyde, while other pollutants studied had an inhibitory effect. Compared with TiO_2_ nanoparticles, TiO_2_ nanotubes may provide higher reactivity and selectivity. Weon, S. et al. [25] synthesized doubly open-ended TiO_2_ nanotubes by potentiostatic anodic oxidation and found higher photocatalytic activity on gaseous acetaldehyde than with TiO_2_ nanotubes and nanoparticles. Yang, W. [56] synthesized cubic SrTiO_3_ (STO) and tetragonal CaTiO_3_ (CTO) with the hydrothermal method to illustrate the effect of a photocatalyst structure on catalytic efficiency. The study found that STO with a cubic structure showed a much higher photocatalytic toluene removal efficiency (about 80.0%) than CTO (about 20.0%), which could be attributed to the promotion of the formation of reactive oxygen species (ROS) and the increasing choice of intermediates. It also improved the ring-opening and mineralization rate.

The photocatalysts supported on the adsorption materials have a higher adsorption capacity, such as TiO_2_, ZnO, ZrO_2_ photocatalyst composites formed by combination with adsorption materials. Fu, X. et al. [57] synthesized TiO_2_/SiO_2_ and TiO_2_/ZrO_2_ nanoparticles with high specific surface area with the sol-gel method. The composite photocatalysts had a higher activity than pure TiO_2_. Mazhar, S.I. et al. [28] used electrospinning technology to synthesize ZnO nanofibers on Teflon with an increased specific surface area. The composite performed a higher adsorption capacity for toluene, acetone, and formaldehyde than ZnO nanoparticles. Natural organic matter and zeolite are also popular in synthesizing photocatalyst composites. Wang, W. et al. [8] provided a green and sustainable method for synthesizing ternary nano-catalysts. Using natural proteins as carbon sources and templates, they synthesized a ternary nano photocatalyst TiO_2_/C/MnO_2_. The photocatalyst showed high selectivity, stability, and excellent photocatalytic performance on the mineralization of a mixture of formaldehyde and toluene under visible light, which was mainly owing to the generation of oxygen vacancies and the distortion of the TiO_2_ lattice. Shojaei, A. et al. [49] synthesized ZnO nanoparticles by a chemical co-precipitation method and coated them on the surface of zeolite to achieve a high removal rate of BTEX.

However, unmodified metal-containing photocatalysts are limited in VOCs decomposition, such as wide energy gap band, deactivation, short electron-hole recombination time, and low VOC destruction efficiency. Therefore, it is necessary to regulate the structure of metal-containing photocatalysts to achieve a higher catalytic activity.

## 3. Doping of the Metal-Containing Photocatalysts

In fact, due to the low concentration and stable structure of VOC molecules in indoor air, some possible photocatalysts are not suitable for removing VOCs. Some commonly used photocatalysts that can only be excited by ultraviolet light, such as TiO_2_ and ZnO, are greatly limited in practical application. From the perspective of the reaction mechanism of the photocatalysts, the band gap energy indicates the relative required energy of promoting the valence band electrons to the vacant conduction band. A narrow band gap requires relatively low energy. Among the common metal-containing photocatalysts, the rutile types of TiO_2_, α-Fe_2_O_3_, BiVO_4_, Ag_3_PO_4_, and CdS have a relatively narrower band gap [13,63], which means they could be excited by visible light. Meanwhile, many current popular photocatalysts are simple in preparation and can degrade most types of VOCs. Despite its advantages, there are some disadvantages such as high carrier recombination rate, restricting its application under visible light or natural sunlight. In addition to developing new photocatalysts, doping is one of the most important modification strategies to adjust the band gap of existing photocatalysts and reduce the carrier recombination rate.

**Figure 1:**
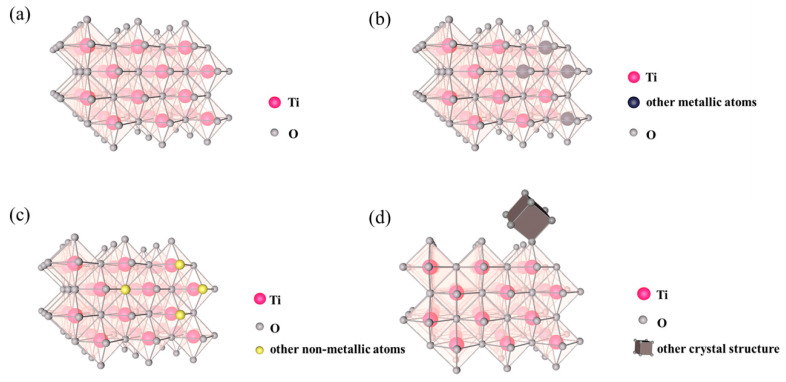
Crystal structure of (**a**) anatase TiO_2_, (**b**) metal doped TiO_2_, (**c**) non-metal doped TiO_2_, and (**d**) other crystal doped TiO_2_.

Contrary to simple physical mixing, photocatalyst doping means that atoms or atomic clusters enter crystal cells to directly replace the original atoms or atomic clusters of the crystal structure and become a part of it. According to the composition of dopants, photocatalyst doping can be classified as metal doping, non-metal doping, co-doping, and other material doping. For example, the crystal structures of anatase TiO_2_ and TiO_2_ with different doping types are shown in Figure 1. As shown in Figure 1a, anatase TiO_2_ has a periodic structure composed of titanium atoms and oxygen atoms with a 1:2 atomic ratio. When doped with metal, such as Ni, Fe, and Al, metal atoms will enter the TiO_2_ crystal structure, replacing a part of the titanium atoms and interacting with oxygen atoms (Figure 1b) and resulting in an atomic ratio of less than 1:2. Similarly, when doped with non-metal (Figure 1c), such as N, C, and S, non-metal atoms will replace oxygen atoms and interact with titanium atoms in a crystal structure, resulting in a ratio of titanium atoms to oxygen atoms greater than 1:2. In addition, the two processes occur simultaneously in co-doping. As shown in Figure 1d, when doped with other crystals, such as Fe_2_O_3_, these crystal units will enter the crystal structure of TiO_2_ as a whole, which is usually achieved by sharing the same atoms or a new chemical bond formation. It should be noted that the location and number of doped atoms are largely random, which depend on the synthesis process of the photocatalyst [36,64].

**Figure 2:**
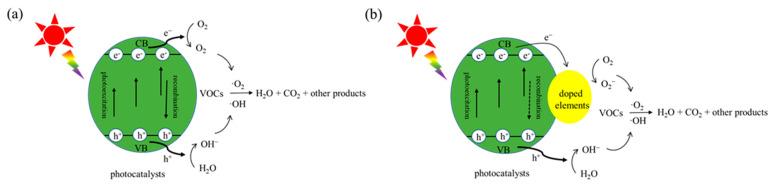
Schematic of photocatalytic removal of VOCs by (**a**) undoped photocatalysts and (**b**) doped photocatalysts.

Over the past few decades, a mass of photocatalysts doped with various materials or elements have been applied in the removal of VOCs, such as N-doped TiO_2_ [65], N, S-doped TiO_2_ [66], Ni-doped SnO_2_/TiO_2_ [67], Fe-doped TiO_2_ [68], and Pt-doped TiO_2_ [69]. Doping will change the original crystal structure, and the introduction of appropriate element or materials can improve the photocatalytic properties and surface structure of the photocatalyst: promote charge transfer, inhibit electron-hole recombination, broaden the light response range, and improve the specific surface area [10] The band gap of the photocatalyst depends on the crystal composition and structure. The formation of photo-generated charge carriers (e^−^, h^+^) is a prerequisite for PCO. However, spontaneous recombination of photo-generated e^−^ and h^+^ results in reduced PCO reactivity (Figure 2a). Doping other materials or elements in the catalysts can weaken the recombination path of photo-generated charge carriers [68,70]. As shown in Figure 2b, doped photocatalysts may generate photo-generated carriers under the excitation of suitable photons, and part of photo-generated electrons could be transferred to the doped materials or atoms and participate in the reduction reaction to remove VOCs. This transfer of electrons weakens the path of recombination and improves the catalytic efficiency [21].

The choice of doping type depends on the characteristics of the photocatalysts and the VOCs’ reaction requirements. For example, TiO_2_ has strong photocatalytic performance under UV, while WO_3_ is a visible light photocatalyst with low catalytic activity due to the serious carrier recombination. Doping WO_3_ on TiO_2_ can create a photocatalyst dopant with increased catalytic ability under visible light [71]. Metal or non-metal elements have similar effects, mainly because they are involved in the formation of catalyst crystals directly. For example, N-doped TiO_2_/WO_3_ has excellent catalytic performance under visible light [70]. In addition, some noble metal elements like Ag may also enhance the light absorption of photocatalysts by surface plasmon resonance [72]. The doping of some transition metal and non-metal elements, such as V, Zr, Cr, Mn, N, etc., may introduce vacancies or defects in the catalyst crystals, and these vacancies or defects often perform as highly active reaction sites in reactions [52]. Co-doping can obtain ternary or multi-element dopants, which are composed of three or more materials or elements and integrate the properties of doped materials and elements to improve the catalytic activity of the photocatalysts.

### 3.1. Metal-Containing Photocatalysts Doped with Metal

**Figure 3:**
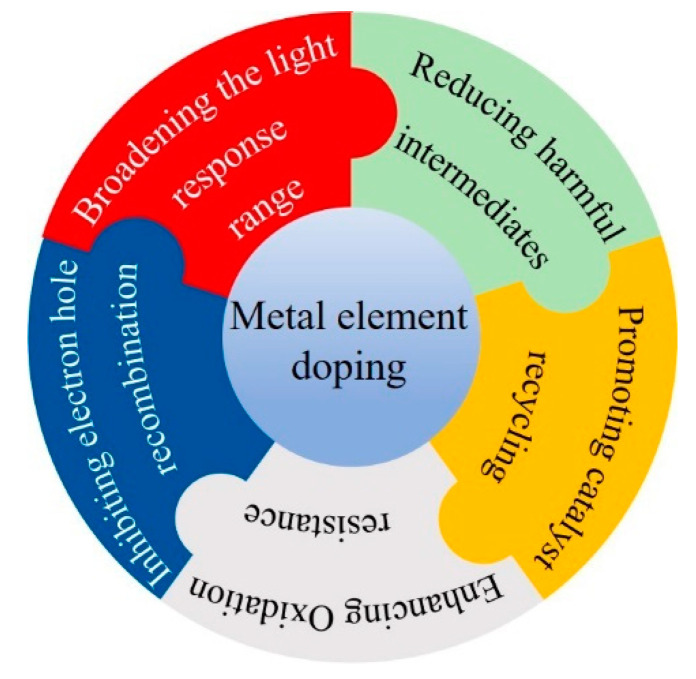
Performance improving by metal element doping.

Coupling metal-containing photocatalysts with metal could enhance a much more complete catalytic oxidation of VOCs into H_2_O and CO_2_ [73]. In particular, metal dopants could extend the light spectrum to visible light, such as V, Cr, Mn, Fe, Co, Ni, or Cu [74]. The addition of metal ion to the photocatalysts can decrease the charge carrier recombination rates and consequently increase the electron-transfer rates. In addition, the introduction of metal can also enhance the stability of the catalyst, reduce the formation of harmful intermediates and promote recycling (Figure 3).

Early in the 1997, Brezova et al. found that doping the metal on the TiO_2_ might significantly increase the photocatalytic activity [12]. The common choices of metal are Li^+^, Zn^2+^, Cd^2+^, Ce^3+^, Co^3+^, Cr^3+^, Fe^3+^, Al^3+^, Mn^2+^, and Pt^0^. However, not all metals have positive effects on metal-doped photocatalysts. Kamat and Meisel [71] found that doping TiO_2_ (5 mol% M^n+^ Ti^4+^) with Co^3+^, Cr^3+^, Ce^3+^, Mn^2+^, Al^3+^, and Fe^3+^ would have a detrimental effect on its photocatalytic activity. Moreover, the valence state and doping amount of metal elements in composite materials also affect the photocatalytic activity of photocatalysts. Therefore, the type and quantity of doping elements are important factors to be considered in the preparation of photocatalysts. According to different properties, metal doping types can be divided into noble metal doping, rare metal doping, and transition metal doping.

#### 3.1.1. Noble Metal and Rare Metal Doping

The most prominent advantages of noble metals are that they are highly resistant to oxidation and corrosion in industrial air, which is with high humid air and has a complex constitution. Additionally, they could also play an important role of trapping and as electron acceptors. Bueno-Alejo C.J. et al. [75] doped ZnO nanostructures with triangular Au through the hydrothermal method. High-resolution electron energy loss spectroscopy (EELS) illustrated the formation of heterojunctions between Au and ZnO, which enhanced the photocatalytic performance across the entire UV/Vis/NIR range. As for the problem of the recovery of nanoparticle photocatalysts, Eun S.R. et al. [72] developed Ag-TiO_2_/Sr_4_Al_14_O_25_: Eu^2+^, Dy^3+^ phosphor beads’ nano photocatalysts for the degradation of toluene by UV/Vis via a sol-gel coating method. The photocatalyst had higher catalytic activity due to the doping of Ag element and was conducive to the recycling of nano materials to prevent secondary pollution. Due to the possible formation of harmful by-products in the photocatalytic process of a variety of VOCs, current studies mainly focus on the photocatalytic degradation of a single species. The doped noble metal elements like Pt can inhibit the production of toxic intermediate by-products in the PCO process. Wu Q. et al. [47] synthesized Pt/TiO_2_ nanoparticles by a modified photo-deposition method. The photocatalytic experiments of formaldehyde-benzene and formaldehyde-xylene showed that the doped Pt could successfully inhibit the formation of toxic methyl phenol. The mechanism analysis revealed that Pt, as a cocatalyst, effectively promoted the activation of O_2_ and guided ·OH to form benzaldehyde from the methyl group, but not from the aromatic ring, and further mineralize into CO_2_.

Rare earth metals are a group of chemical elements, including scandium (Sc), yttrium (Y), and another 15 lanthanides. For rare earth metals, their special feature is electron distribution, which has 4f and empty 5d orbitals. In addition, the photocatalysts with rare earth metals could also enhance the absorption of VOCs. Burns et al. [76] proposed that doping photocatalysts with rare earth would twist the crystal lattice because of the difference of the ionic radius. Yurtsever H.A. [77] also proposed that rare earth ions could prevent the anatase phase (TiO_2_) transiting into the rutile phase (TiO_2_). Zhu et al. [78] synthesized Cd, Y-co-doped rutile TiO_2_ nanorod arrays and found that doping Y into TiO_2_ could improve the electron transport channel and increase the carrier concentration.

#### 3.1.2. Transition Metal Doping

Transition metals are commonly less expensive than noble metals. The most popular transition metals studied are Mn, Fe, Cu, V, Ni, etc., which contribute to the reduction of the energy band gap, decreasing the recombination rate of electron-hole pairs, and extending the absorption of the solar spectrum [20,79]. The amount of dopant for transition metals has an optimum level. Only when the amount of metal dopant is at a reasonable level, it can become a charge carrier bridge, decreasing the recombination rate of the electron-hole. If the amount of metal dopant exceeds the optical level, it can also play a role as a recombination site [20]. Additionally, the type of dopants also contributes to the performance of VOCs’ decomposition [77].

The most popular choices of transition metal are iron (Fe) and nickel (Ni). Doping Fe for the photocatalysts is an effective way to narrow the energy band, which can extend the light absorption range into visible light. The significant mechanism that Fe promotes with the photocatalysts’ performance is the Fe^3+^/Fe^2+^ mutual conversion process, which inhibits the recombination of charge carriers. Dong et al. [80] proposed that the Fe^3+^ accepted photo-generated electrons, then transferred them to O_2_, and at last produced O_2_^−^, which is a strong oxidant. Additionally, Fe^3+^ is easily added into the TiO_2_ crystal lattice due to the similar radius of Fe^3+^ (0.64 Å) and Ti^4+^ (0.68 Å). Humidity is an important factor affecting VOCs oxidation reactions, because water molecules act as the electron acceptor and complete the adsorption on the surface of the photocatalysts. Saqlain S. et al. [81] studied the effect of gaseous water in the air on the photocatalytic removal of acetaldehyde and toluene by Fe-doped TiO_2_ nanoparticles prepared via a chemical meteorological deposition method. The results indicated that the removal rate of the pollutants increased firstly and then decreased with the increase of humidity, but the optimum humidity varied with VOC species. Moreover, Shayegan Z. et al. [50] reduced the surface hydrophilicity of a Fe-doped TiO_2_ catalyst by surface fluorination and obtained a photocatalyst with higher catalytic activity for the removal of VOCs. In terms of Ni, it could also extend the solar absorption spectrum and decrease the recombination. However, the mechanism is not quite the same. The Ni^2+^/Ni^+^ mutual conversion process could trap the electrons from recombination with photo-excited holes and transfer it to O_2_. Ni^+^ could also react with Ti^4+^ to generate Ni^2+^ and Ti^3+^ [82]. Huang et al. [83] found that the different performance of TiO_2_ catalysts doped with different metal dopants and transition metals could block TiO_2_ pores and decrease the active surface area. In addition to the common transition metal elements used for doping, the doping of uranium (U) can also broaden the light response range of TiO_2_ to visible light and significantly improve photocatalytic activity, but its radioactivity and high price limit further application. In addition, Ir-doped TiO_2_ also showed excellent performance in the photocatalytic degradation of toluene [84].

### 3.2. Metal-Containing Photocatalysts Doped with Non-Metal

Other than metal dopants (also considered as cation dopants), recently, non-metal dopants are considered more suitable for narrowing the energy gap band [85]. The common choices of non-metal dopants are N, C, S, B F, etc. The basic principle for non-metal dopants narrowing the energy gap band is that dopant states are slightly above the valence band edge, rather than acting as a substitute of charge carriers. In addition, the non-metal dopants could also substitute oxygen in the semiconductor lattice, which would also be responsible for expanding the adsorption of the solar spectrum to visible light due to the p-orbitals. Some researchers believe that non-metals optimize photocatalysts by mainly changing its morphology and composition, which could significantly increase the degradation ability of VOCs [86]. The most popular non-metal dopant photocatalyst types are N-doped and C-doped.

#### 3.2.1. N-Doped

Various N-doped TiO_2_ nanomaterials synthesized by the hydrothermal method were studied in degrading VOCs, such as formaldehyde, acetaldehyde [87], acetone [88], ethyl benzene, o, m, p-xylenes [85], and toluene [68]. N can effectively reduce the band gap of TiO_2_. According to Asahi et al. [88], TiO_2_-xNx has active wavelengths less than 500 nm, which contain the main portion of the solar light (460 nm).

**Figure 4:**
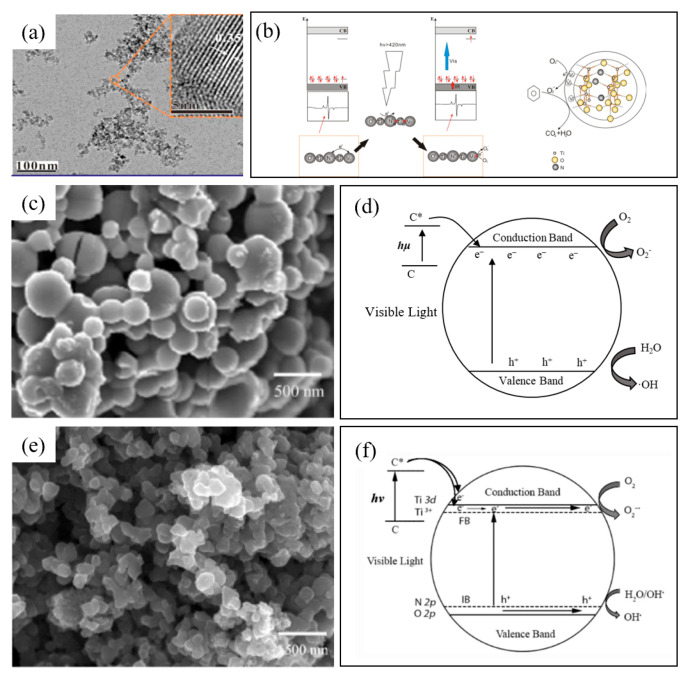
Images and photocatalytic mechanisms of non-metal (co-)doped photocatalysts: (**a**) TEM image of N-doped TiO_2_ nanoparticles; (**b**) electron transfer path and pollutant degradation mechanism of N-doped TiO_2_ (reproduced with permission [89], copyright 2015, Elsevier); (**c**) SEM image of C-doped TiO_2_ nanoparticles; (**d**) photocatalytic mechanism of C-doped TiO_2_, C* represents photosensitized C (same as below); (**e**) SEM image of C, N co-doped TiO_2_ nanoparticles; (**f**) photocatalytic mechanism of C, N co-doped TiO_2_ (reused with permission [90], copyright 2015, Springer).

Furthermore, N-doped methods change the physical features of the substrate photocatalysts, including hardness, electrical conduct ability, refraction index, and photocatalysis in the visible light region [20,88]. A characteristic of N-doped materials is that N can enter the TiO_2_ lattice and other similar-lattice photocatalysts, owing to the similar radius of N and oxygen. Unlike the metal-doped type, adding an additional energy state into the energy band gap, N-doped type levels up the 2p-orbital of the valence band and leads to a narrower energy band gap [89]. Totsaporn et al. [65] prepared a series of N–TiO_2_ photocatalysts via the hydrothermal method and employed urea compounds as nitrogen sources and found that the increase of the nitrogen dopant enhanced the anatase phase strength and delayed the phase transformation from anatase to rutile. The N was incorporated into the TiO_2_ lattice by nitrogen atom substitution and/or interstitial sites. In the earlier study, Albrbar et al. [66] proposed two types of N-doped materials on photocatalysts. One was interstitial N^3−^ anion and the other was substituting O^2−^ anions. Both could narrow the energy band gap, and the former created a correlated interaction between N 2p and Ti 3d orbitals. Zeng et al. [89] obtained N-doped TiO_2_ nanoparticles with a particle size of about 5 nm by annealing in ammonia (Figure 4a). They found that oxygen vacancies were introduced with nitrogen doping. As shown in Figure 4b, the doped nitrogen and oxygen vacancies in the lattice made the catalyst show high catalytic efficiency for benzene under visible light.

#### 3.2.2. C-Doped

C-doped metal-containing photocatalysts, for instance, C-doped TiO_2_, can inhibit the transition from rutile to anatase, and significantly enhance the adsorption ability of VOCs. It can also improve the degradation efficiency by accelerating the transfer rate of charge diffusion in the conduction band [90]. A detailed comparison of C- and N-doped TiO_2_ photocatalysts was carried out [90]. As shown in Figure 4c, the diameter of C-doped TiO_2_ nanoparticles becomes larger, because unlike nitrogen atoms, C does not enter the crystal of TiO_2_, but bounds to the surface of TiO_2_ nanoparticles in the form of substances. The incorporation of carbon enhances the adsorption capacity, while the photosensitized carbon promotes the electron transfer of the photocatalysts (Figure 4d). Ridha et al. developed the anatase TiO_2_ composed of Activated Carbon-TiO_2_ (AC-TiO_2_), Olive Pits-TiO_2_ (OP-TiO_2_), and Wood Shaving-TiO_2_ (WS-TiO_2_) through an ultrasonic-assisted sol-gel process and enhanced the efficiency of photocatalytic reduction by narrowing the band gap of TiO_2_ nanoparticle deposited in the carbonaceous materials, forming the photosensitizer action of carbonaceous materials passing Ti-O-C bonds and transferring electrons from TiO_2_ to carbonaceous materials. However, whether the doped C is substitutional or interstitial is still under debate. Di Valentin et al. reported a similar doped type of C, which was similar to the N-doped type [91]. In addition, in the calcination procedure, there will remain some residual carbon-containing species, which can also narrow the energy gap band; however, with complicated compositions [86].

Previous studies have pointed out that carbon quantum dots (CQDs)-modified semiconductors are promising photocatalysts and can be used as sustainable environmental pollutant-purification materials. Paszkiewicz-Gawron M. et al. [55] synthesized spherical, rod-shaped, and faceted nanomaterials with a wide energy band perovskite doped by CQDs, graphene quantum dots, Er: SrTiO_3_ (titanate), SrSnO_3_ (stannate), and AgTaO_3_ (tantalate) via thermal the solvent method, which were used to study the degradation of toluene. They found that the doping of carbon graphene quantum dots, Er, and CQD could enhance the light absorption of photocatalysts.

### 3.3. Metal-Cointaining Photocatalysts Doped with Co-Dopants

Even though a single dopant can significantly promote substrate metal-containing photocatalysts, there are still some limitations of single-element optimization. Therefore, more researchers have concentrated on co-doping to compensate for these drawbacks. There are mainly three types of co-dopants, including metal/non-metal and non-metal/non-metal. However, the mechanism of co-doping improving photocatalytic activity is not completely explained because of the unclear interaction between different elements [92].

#### 3.3.1. Metal/Metal Co-Dopants

**Figure 5:**
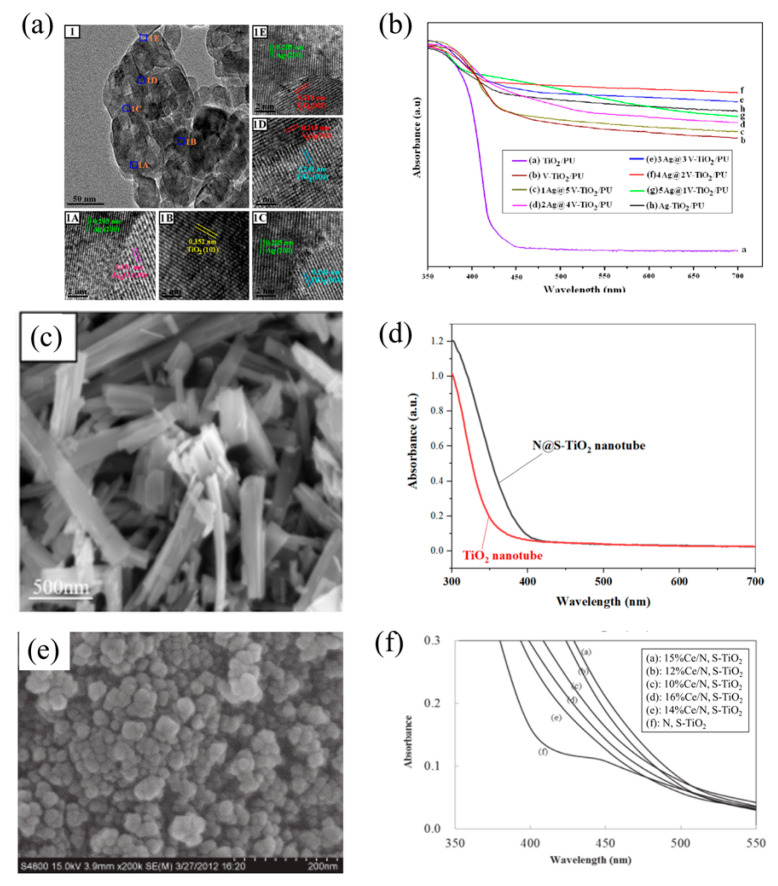
Images and UV–Vis absorption spectra of co-doped photocatalysts: (**a**) TEM image of Ag, V-doped TiO_2_ nanoparticles; (**b**) UV–Vis absorption spectra of Ag, V-doped TiO_2_ with different doping ratios (reused with permission [92], copyright 2016, Elsevier); (**c**) SEM image of N, S co-doped TiO_2_ nanotubes; (**d**) UV–Vis absorption spectra of N, S co-doped TiO_2_ (reproduced with permission [94], copyright 2020, Springer); (**e**) SEM image of Ce, N, S co-doped TiO_2_ nanoparticles; (**f**) UV–Vis absorption spectra of Ce, N, S co-doped TiO_2_ (reused with permission [95], copyright 2016, Elsevier).

Basically, a metal/metal co-dopant type has two main functions to promote the degradation performance of VOCs. One of which is to help disperse novel metal uniformly on the photocatalysts; the other is to help form oxygen vacancies. Zeng et al. [89] synthesized Ag, V co-doped TiO_2_ nanoparticles with a novel modular calcination method. As shown in Figure 5a,b, Ag, V co-doped TiO_2_ nanoparticles have a particle size of about 30 nm and show strong visible light-absorption ability. There are some pieces of research that transformed the performance by affecting other structures. Andrade Neto et al. [93] obtained ZnO nanoparticles co-doped with Fe^3+^ and Pb^2+^ via the hydrothermal method, and found that with the increase of doping materials, the morphology of the nanoparticles lost consistency and the size of the nanoparticles was reduced.

Zhang et al. [96] found that when adding Li^+^, Na^+^, and K^+^ to Pt/TiO_2_ catalysts, Pt would be dispersed much more uniformly than pure Pt/TiO_2_, and that such adjustment was automatic. Pham [92] also found a similar phenomenon when studying the performance of Ag-V-TiO_2_/PU, in which the distribution of Ag/V were uniform, and proposed the mechanisms in terms of the increase of oxygen vacancies (Ti^3+^ formation): when Ag/V were incorporated on the TiO_2_, the ratio of Ti^3+^/Ti^4+^ would increase, indicating a better degradation performance.

#### 3.3.2. Non-Metal/Non-Metal Co-Dopants

Non-Metals co-doped photocatalysts are considered to have a function of reinforcing the absorption of visible light and have degradation abilities [87]. Previous research indicates the mechanism of co-dopants optimize TiO_2_, including C, N, O, S, and F. Jirapat et al. [90] synthesized C, N co-doped TiO_2_ nanoparticles via a sol-gel method (Figure 4e). They pointed out that C, N co-doping did not change the morphology of TiO_2_, but co-doping could produce a synergistic effect to improve activity, which was attributed to the reduction of the Eg by N-doping and the photosensitization effect of C-doping. Li et al. [97] indicated that the N dopant was mainly responsible for enhancing visible light absorption by increasing the formation of superoxide (O_2_^−^) radicals through oxygen vacancy sites. However, F dopant helps to create new active sites and hydroxyl radicals (·OH). Therefore, N, F co-doped TiO_2_ shows a great ability in degrading gas-phase acetaldehyde, toluene, and trichloroethylene. Additionally, S, N co-doped photocatalyst also exhibited an extension of absorption in visible light [20,94]. Yen et al. [94] synthesized N, S co-doped TiO_2_ nanotubes via a sol-gel combined hydrothermal method (Figure 5c). The UV–Vis absorption spectra of N, S co-doped TiO_2_ in Figure 5e indicates a stronger visible light response and lower Eg. Another piece of research by Li et al. [98] reported Ti-O-N, Ti-N, Ti-O-S, and Ti-O-C, four types of bonds in the N, S, C-TiO_2_ photocatalysts, which strengthened the UV and visible light absorption. Besides, Dong et al. [87] reported that, in the N-doped photocatalyst, two nitrogen atoms had replaced three oxygen atoms to balance the neutrality, which produced an oxygen vacancy in the TiO_2_ crystal lattice. Oxygen vacancy could be an active site to form superoxide radicals (O_2_^−^).

#### 3.3.3. Non-Metal/Metal Co-Dopants

Logically, since non-metal and metal dopants have an individual function of optimizing photocatalysts, a great deal of researchers added both non-metal and metal dopants on the photocatalysts to modify them. Such co-pants can effectively increase the inter-facial charge rates and decrease the recombination rate of the electron-hole. Furthermore, metal dopants provide a substitute level nearly above the conduction band, and non-metal prefers to form a new level closest to the valence band, which indicates the extension of absorption of visible light, enhancing photocatalytic activity and decreasing the recombination rate of the electron hole [80]. Lee et al. [95] synthesized Ce N S co-doped TiO_2_ nanoparticles with different mass ratios by the incipient wet impregnation method (Figure 5e). As shown in Figure 5f, UV–Vis absorption spectra shows that the utilization rate of visible light varies with the doping ratio of TiO_2_. Besides, Dong et al. [80] found that the photo-degradation efficiency was improved to 97% when N and Fe were both doped on TiO_2_. Fe-ion played a role by modifying the surface of N-TiO_2_, which improved the absorption ability under both UV and visible light spectrum. Sirivallop, A. et al. [99] used N/Ag co-doped TiO_2_ photocatalyst synthesized by the solvothermal method to catalyze the degradation of gaseous methanol under visible light. TiO_2_ is inactive under visible light, while the N/Ag co-doped TiO_2_ photocatalyst shows high quantum yield and methanol conversion as a result of joint influence by Ag surface plasmon resonance, the effects of Ag and N on the band gap of TiO_2_, and their effects on particle aggregation and photocatalyst acidity.

### 3.4. Metal-Cointaining Photocatalysts Doped with Other Materials

**Figure 6:**
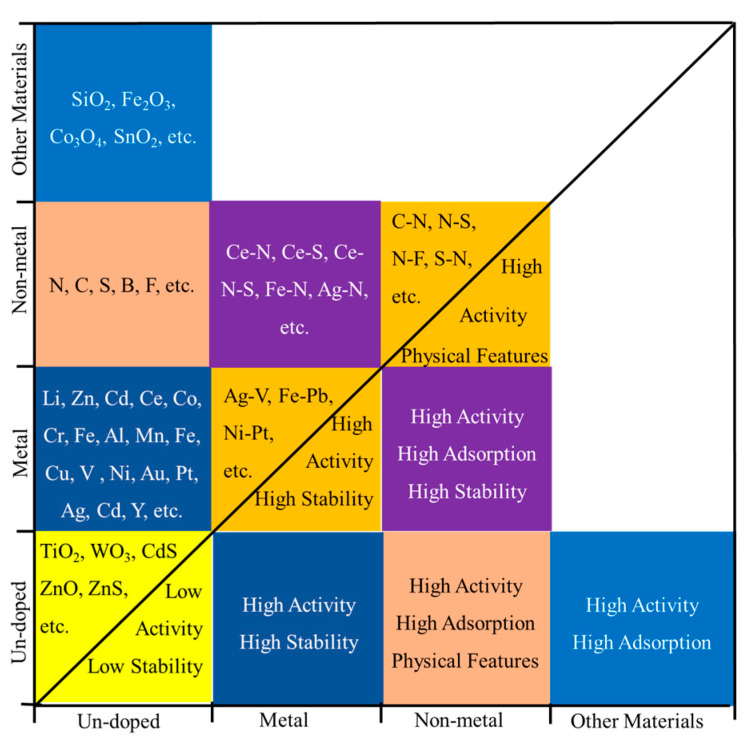
Metal, nonmetal, and other materials used for (co-)doping and the performance of dopants. Each square represents one doping type (the first square represents undoped photocatalysts), squares of the same color represent the same doping type, the upper-left part of the figure represents the (co-)doped elements/materials, and the lower-right part represents the performance.

The doping of metal-containing photocatalysts and other materials is generally with metal oxide or a different phase of photocatalysts. As shown in Figure 6, the doping of materials can effectively promote the light absorption and utilization, and improve the specific surface area of the photocatalysts. Bani Sharif et al. [100] synthesized Fe_2_O_3_-doped TiO_2_ with different doping ratios via an ultrasonic-assisted coprecipitate method, and the Fe_2_O_3_-doped TiO_2_ showed a trichloroethylene removal efficiency about 200% higher than that of commercial P25, which was attributed to the reduction of band gap energy and the improvement of specific surface area. Coupling pure metal-containing photocatalysts, TiO_2_ for instance, with a smaller band gap semiconductor could extend light utilization to visible light [74]. Pan et al. [101] doped a range of WO_3_ ratios on the mesoporous anatase TiO_2_, and the prepared mesoporous WO_3_/TiO_2_ nanomaterial could greatly increase the utilization of the light spectrum, and extend the range from UV to visible light. Unfortunately, in the meantime, when the absorption of visible light increased, the photocatalytic activity in the UV decreased.

Jian et al. [102] successfully synthesized a new type of Co_3_O_4_/CeO_2_-Co_3_O_4_-graded binary oxide by using carbon spheres as the hard templates, which had high stability and excellent catalytic oxidation activity for CH_2_Br_2_. The rich high-valence Co of the material promoted the oxidation of CH_2_Br_2_. Between Co_3_O_4_ and CeO_2_ of Co_3_O_4_/CeO_2_-Co_3_O_4_ (HS), there was a special hierarchical porous structure, strong synergistic interaction, and high specific surface area, which was also conducive to the catalytic oxidation process.

In addition, composing other materials could also decrease the rate of electron-hole recombination. Among the various materials added to the pure photocatalysts, SnO_2_ and TiO_2_ are a popular combination due to their similar lattice parameters and geometry structure (tetra-goal structure). When the TiO_2_ and SnO_2_ are combined, TiO_2_ photo-generated electrons could move to the SnO_2_ conduction band, thus, the electrons and holes are restricted from recombination [67]. However, the interesting part of these two photocatalysts combination is that the energy gap band of SnO_2_ (3.5 eV) is bigger than that of TiO_2_ (3.0 eV), therefore, resulting in a blue shift in TiO_2_ solar spectrum, which indicates the limitation of TiO_2_ absorption of visible light. Roman Khan and Tae-Jeong Kim added Ni^2+^ into TiO_2_-SnO_2_ and proposed that xNi-TiO_2_-SnO_2_ were active under visible light [69]. CdS-TiO_2_ combination is also considered to reduce the recombination rate of charge carriers. Under visible light illumination, CdS with a relatively lower energy gap band (2.4 eV) could photo-generate electrons and holes and the excited electrons could move to the conduction band of TiO_2_ [67].

In terms of preventing photocatalyst deactivation, early research has shown that the SiO_2_/TiO_2_ photocatalyst has the function of slowing down the deactivation rate [103]. Rafael and Nelson [103] proposed that it might be because of the increased Brùnsted acidity on the surface. The SiO_2_/TiO_2_ photocatalyst showed optimized performance when decomposing the benzaldehyde.

Some studies have also concentrated on TiO_2_/SiO_2_. Yu et al. [104] proposed that if increasing the SiO_2_ percentages would increase the surface hydroxyl, it could increase the decomposition efficiency of pollutants. The reason is that the surface hydroxyl is responsible for reaping holes in the valence band, further preventing the recombination of electron-holes. However, the high percentage of the SiO_2_ might adversely decrease the efficiency of decomposition. Yu et al. [104] claimed that the percentage of SiO_2_ could not be higher than 10 mol%. Zhan et al. [73] reported that increased percentage of SiO_2_ in the TiO_2_/SiO_2_ composition might enhance the specific surface area of photocatalysts. However, similarly, an excessive amount of silica ratio in TiO_2_/SiO_2_ would decrease the performance of the destruction of reactants because the charge carriers might recombine in a higher rate.

## 4. Conclusions and Perspectives

In this article, the different doping types and mechanisms of nano metal-containing photocatalysts for the removal of VOCs are reviewed. The most common photocatalysts used for the removal of VOCs are a class of semiconductor metal-containing catalysts, including oxides and binary chalcogenide metal semiconductors. Based on the principle that photocatalysts generate carriers to form free radicals and ROS under UV/Vis light, VOCs are oxidized and degraded into CO_2_, H_2_O, and other products. Metal-containing photocatalysts with nanostructures and different doping types show more attractive application prospects. Nanostructure photocatalysts with high activity such as nanoparticles, nanotubes, nanobelts, and nanofibers are widely researched in the laboratory and in practical applications, which can adjust the crystal structure of the photocatalysts, increase the specific surface area, and provide more reaction sites. Common doping types include metal doping, non-metal doping co-doping, and other material doping. Doped photocatalysts usually have a wider range of light response and improved photocatalytic activity. Element doping is mainly based on the optimized crystal structure to improve the activity of the photocatalysts, and some material doping can also improve the adsorption capacity of the catalysts. This work will provide a reference for selecting appropriate doping strategies to develop and modify new photocatalysts.

Nano metal-containing photocatalysts have been developed vigorously in the past few years. However, there are still few visible light-responsive efficient and stable photocatalysts. In addition, the recovery of metals containing catalysts and secondary pollution also needs attention. In the future, PCO will be applied on a large scale in industry and life to remove VOCs, and the development of new low-cost, environment friendly, and efficient photocatalysts will become a development trend in the next few decades.

## Figures and Tables

**Table 1:** The energy band gap of common metal-containing photocatalysts [37,38,39,40,41,42,43,44,45].

Catalyst	Eg ^1^ (eV)
TiO_2_ rutile	3.02
TiO_2_ anatase	3.23
ZrO_2_	5.00
ZnS	3.76
SnO_2_	3.65
ZnO	3.37
CeO_2_	3.18
WO_3_	2.75
CdS	2.47
α-Fe_2_O_3_	2.09

^1^ Eg is the energy band gap.

**Table 2:** Some nano metal-containing photocatalysts for the removal of VOCs.

Catalyst	Nanostructure	Synthetic Method	Degraded VOC	Ref
P25 *	nanoparticles	flame spray pyrolysis	formaldehyde	[18]
P25 *	nanoparticles	flame spray pyrolysis	formaldehyde	[51]
TiO_2_	nanoparticles	sol-gel technique	TCE	[48]
TiO_2_	nanotube	potentiostatic anodic oxidation	formaldehyde/toluene	[25]
TiO_2_	nanotube	hydrothermal method	methanol/n-hexane	[26]
TiO_2_	nanotube	hydrothermal method	TCE	[27]
CeO_2_	nanoparticle	redox and steam treatment	n-hexane/cyclohexane	[52]
ZrO_2_	mesoporous	hydrothermal method	chlorobenzene	[53]
WO_3_	nanofilm	impregnation method	acetaldehyde	[54]
SrTiO_3_	nanoparticles	hydrothermal method	toluene	[55]
SrTiO_3_	nanoparticles	hydrothermal method	toluene	[56]
CaTiO_3_	nanoparticles	hydrothermal method	toluene	[56]
SrSnO_3_	nanoparticles	hydrothermal method	toluene	[55]
AgTaO_3_	nanoparticles	hydrothermal method	toluene	[55]
TiO_2_/CdS	nanobelts	successive ionic layer adsorption and reaction method	toluene	[29]
TiO_2_/CdS	nanofiber	anodic oxidation.	toluene	[30]
TiO_2_/ZrO_2_	nanoparticles	sol-gel technique	ethylene	[57]
TiO_2_/SiO_2_	nanoparticles	sol-gel technique	ethylene	[57]
TiO_2_/SiO_2_	nanoparticles	hydrolysis	toluene	[58]
TiO_2_/SiO_2_	nanoparticles	impregnation method	acetaldehyde	[59]
TiO_2_/ZrO_2_	nanofilms	sol-gel technique	acetone	[60]
TiO_2_/Sr_2_CeO_4_	nanoparticles	grinding and heating	benzene	[61]
TiO_2_/C/MnO_2_	nanoparticles	solvothermal method	formaldehyde/toluene	[8]
ZnO/PTFE	nanofibers	electrospinning	toluene/formaldehyde/acetone	[28]
ZnO/zeolite	nanoparticles	chemical co-precipitation	benzene series	[49]
ZrO_2_-SiO_2_	nanoparticles	atomic layer deposition method	benzene	[62]

* P25 is a commercial nano-TiO_2_ powder. TCE and PTFE refer to trichloroethylene and Teflon, respectively.
